# Searching for Radial Symmetry

**DOI:** 10.1177/2041669517725758

**Published:** 2017-08-22

**Authors:** Ben J. Jennings, Frederick A. A. Kingdom

**Affiliations:** McGill Vision Research – McGill University Department of Ophthalmology, Montreal General Hospital, Canada

**Keywords:** Mirror, radial, spatial vision, symmetry, visual search

## Abstract

Symmetry is ubiquitous in the natural world. Numerous investigations, dating back over one hundred years, have explored the visual processing of symmetry. However, these studies have been concerned with mirror symmetry, overlooking radial (or rotational) symmetry, which is also prevalent in nature. Using a visual search paradigm, which approximates the everyday task of searching for an object embedded in background clutter, we have measured how quickly and how accurately human observers detect radially symmetric dot patterns. Performance was compared with mirror symmetry. We found that with orders of radial symmetry greater than 5, radial symmetry can be detected more easily than mirror symmetry, revealing for the first time that radial symmetry is a salient property of objects for human vision.

## Introduction

**Figure 1.**
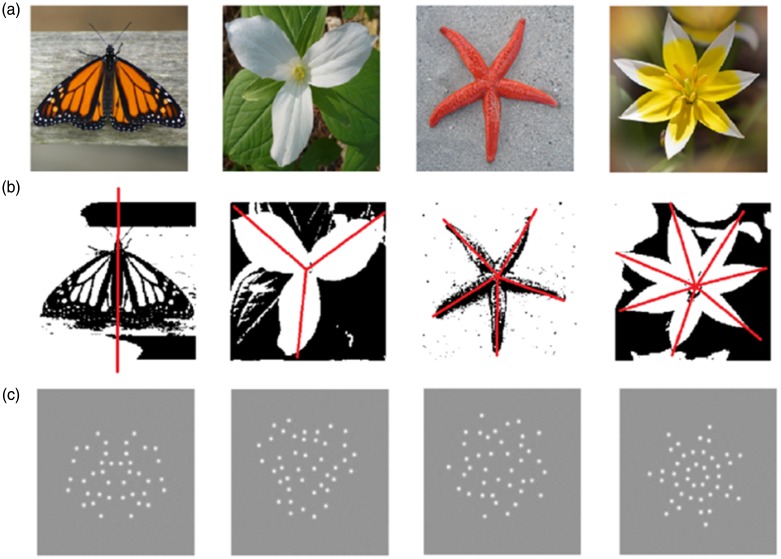
(a) Examples of symmetric objects found in nature. From left to right, a mirror-symmetric monarch butterfly (*Danaus plexippus*), three different orders of radial symmetry, first a white wake-robin flower (*Trillium grandiflorum*) exhibiting third-order radial symmetry, a starfish (*Fromia milleporella*) exhibiting fifth-order radial symmetry and a multiflowering tulip (*Tulipa tarda*) exhibiting seventh-order radial symmetry. (b) Images from (a) after thresholding with their axis/axes of symmetry indicated by red lines. (c) Examples of the symmetric target patches employed in our search task that correspond to the symmetry type above.

Symmetric structures exist throughout the biological world. The visual perception of symmetry is an important biological function, enabling animals to detect the presence and type of a variety of biological objects in the scene. The most studied type of symmetry in visual perception is mirror symmetry (Barlow & Reeves, 1979; Machilsen, Pauwels, & Wagemans, 2009; Rainville & Kingdom, 2000; Sasaki, Vanduffel, Knutsen, Tyler, & Tootell, 2005; Scheib, Gangestad, & Thornhill, 1999; Treder, 2010), as exemplified by the butterfly in Figure 1(a). While two electrophysiological studies, using event-related potentials, have revealed that humans are sensitive to both mirror and radial symmetry (Bertamini & Makin, 2014; Makin, Wilton, Pecchinenda, & Bertamini, 2012), radial symmetry (also referred to as rotational symmetry) has not been systematically studied in a visual search paradigm. This in spite of the fact that like mirror symmetry, radial symmetry is ubiquitous in nature: The flowers and starfish in Figure 1 are exemplars. In this communication, we measured the ability of humans to visually detect a radially symmetric pattern and compared our results with the detection of mirror symmetry.

An object is said to exhibit radial symmetry if it is invariant to being rotated though a given set of angles. For example, the three petal flower in Figure 1(a) possesses third-order radial symmetry as it is composed of three similar repeating sectors, allowing it to be rotated around its origin in multiples of 120° with little or no change to its visible structure. The starfish and yellow flower in Figure 1(a) exhibit fifth- and seventh-order radial symmetry respectively, allowing rotations in multiples of 72° and 51° with little or no change to its visible structure. This order property of radial symmetry raises the question: Are some orders of radial symmetry more visually detectable than others, especially in cluttered environments, as in the natural visual world? In the current study, we measure visual search times as a function of radial symmetry order, that is, fold level, for these two classes of symmetry.

A previous study on mirror symmetry hints at what one might expect when varying the order of radial symmetry (Wenderoth & Welsh, 1998). Mirror symmetry is produced when an object or pattern is reflected across an axis. Although mirror-symmetric patterns in nature typically have just one axis of symmetry (e.g., the butterfly in Figure 1), it is possible to create patterns with more than one mirror-symmetric axis. Wenderoth and Welsh (1998) investigated the effect of adding more mirror-symmetric axes to a target pattern (dot patterns and solid shapes). They found that performance (correctly identifying a symmetric stimulus) improved when the stimuli contained more than one mirror-symmetric axis. Adding more axes to a radial-symmetric pattern, however, is a different manipulation from adding more axes to a mirror-symmetric pattern: With radial symmetry, the repeating sectors are positioned via a rotation around the origin, whereas with mirror symmetry, the pattern either side of each additional axis is mirror symmetric.

We measured search times for observers to detect a radially symmetric dot pattern among random dot distractor patterns as a function of the order of radial symmetry. For comparison, we also measured search times for mirror-symmetric patterns.

## Methods

### Observers

Ten observers with normal visual acuity, after providing informed consent, participated in the experiment. The Research Ethics Board of the McGill University Health Centre gave prior approval for the psychophysical testing.

### Apparatus

Custom software, employing PsychToolbox (Pelli, 1997) functions, was used to generate the stimulus which was displayed on a ViewSonic monitor, driven at 60 Hz with a resolution of 1920 × 1080 pixels (the viewing distance was fixed at 70 cm).

### Stimuli

The target patches were composed of Gaussian blobs, defined as luminance increments relative to the uniform mid-grey background. The standard deviation of the Gaussian was 0.027°, but the blobs were gated to a diameter of 3.8°.

In all conditions, the positions of the blobs were quasi-random, meaning that their positions were random but with the constraint that there was no overlap between them. The target patches were created according to the following rules. The radially symmetric targets were produced by defining a sector subtending an angle corresponding to the order of the symmetry, for example, third order corresponded to 120° (i.e., 360/order). Within this region, the blobs were quasi-randomly positioned. This sector was then duplicated for the remaining sectors of the target’s circular area. Three radial orders were employed, third, fifth and seventh, corresponding to three sectors of 120°, five sectors of 72° and seven sectors of 51°, respectively. The mirror-symmetric axis and the first sector in the radial patterns were always aligned vertically, so as to minimise effects due to orientation (Royer, 1981). The mirror-symmetric targets were created by arranging half of the blobs quasi-randomly within the left-hand semicircular region of the target area, and reflecting the blob coordinates around the central ‘mirror’ axis, so that the condition *f*(*x*,*y*) = *f*(−*x*,*y*) was satisfied. Examples of all the symmetry conditions are illustrated in Figure 1(c) – panels from left to right contain mirror followed by radial third, fifth and seventh. New target patches were generated for each trial so no two trials contained the same target. Distractor patches comprised solely quasi-randomly positioned blobs and were confined to similar circular regions as the targets. Each target and distractor comprised 42 blobs, with the one exception being the radial fifth-order targets which were composed of 40 blobs, due to 42 not dividing exactly into the five sectors.

**Figure 2.**
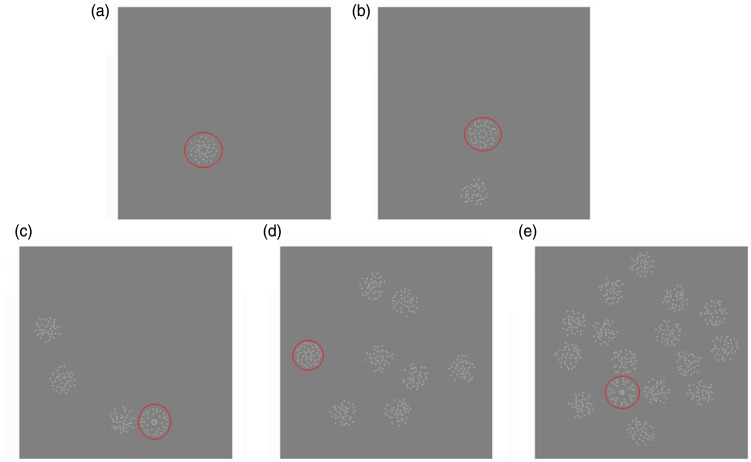
Examples of target present search arrays for set sizes: (a) 1, (b) 2, (c) 4, (d) 8 and (e) 16. Target patches are highlighted for illustration purposes only by the red circles not visible during the experiment.

The search arrays were created by positioning the target and distractor patches randomly within a larger circular region with a diameter subtending 26.3°. The set sizes tested were 1, 2, 4, 8 and 16 patches. On the target trials, one of the set was a target while the remaining number were distractors, while on the no-target trials the whole set were distractors. Figure 2(a) to (e) shows examples of the five set sizes for the target present condition (seventh-order radial target), the red circles (for illustration only) indicate the target patches.

### Procedure

In a typical visual search task (Treisman & Garry, 1980), observers are required to search for a target among a number of distractors. On half the trials, the target is present, and on half the target is absent. On each trial, the observer indicates whether the target is either present or absent, and the time to reach the decision – the reaction time (RT) – is recorded along with the decision. This is repeated for trials with different numbers of distractors.

Each trial of the search task followed the same time course. A fixation cross was first displayed in the centre of the screen for 1 s, followed by the search array. The observer then searched (eye movements were allowed) the display and submitted their response via a key press indicating whether the target patch was present or absent. Observers were instructed to respond as quickly and accurately as possible. The fixation cross would then reappear, and the observer was required to re-direct their gaze to the cross before the next trial commenced. There was a 50% chance on a given trial that the display would contain a target. The experiment followed a block design meaning that within a block the target was the same type of symmetry. The block order was counterbalanced between observers. The button assignment was also counterbalanced between observers meaning that different fingers were used to indicate target-present and target-absent responses. Two blocks, each containing 100 trials, were conducted for each target type, resulting in 200 trials per condition per observer (40 trials per set size; 20 present and 20 absent). For each condition, the RT and accuracy were calculated for both target-present and target-absent trials, and the data collapsed across observers.

Traditionally, the pattern of RTs as a function of set size has been used to distinguish between ‘parallel’ and ‘serial’ search. Parallel search is said to occur when search times do not increase with RT because observers do not need to search though the items one at a time. In other words, the target is located pre-attentively, or ‘pops out’ (Thornton & Gilden, 2007). On the other hand if RTs are found to increase with set size, this implies a serial (one at a time) search. Although there has been some debate in the literature regarding whether search is truly parallel or serial from the analysis of RTs as a function of set size (Kristjansson, 2015; Wolfe, 2016), calculating the number of items processed per second provides a simple metric for comparing search efficiency, and these are presented in the Results section.

To summarize, the present study measured performance in a visual search task for three different orders of radial symmetry as well as for mirror symmetry. Performance was measured for each type of symmetry as function of the number of distractor patches.

## Results

**Figure 3.**
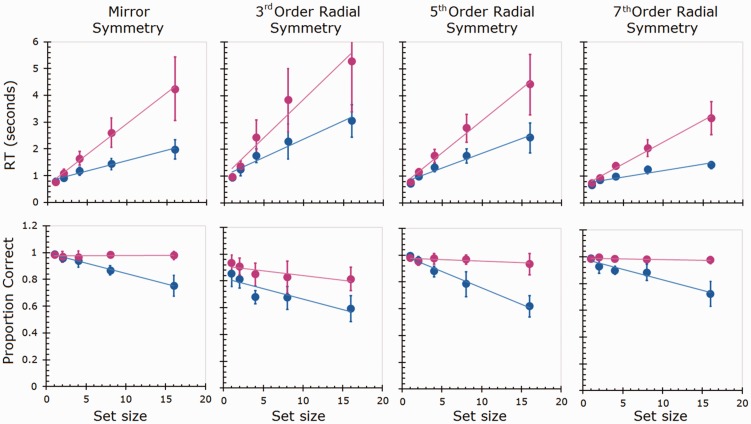
The top row plots the RTs for the four symmetry types; the bottom row plots the corresponding proportion correct data. The blue data points represent the target present condition; the magenta data points represent the target absent condition. All error bars are ±2 standard errors. RT = reaction time.

Figure 3 plots median RTs (top row) and proportion correct (bottom row) data for the four experimental conditions. Left to right, the panels are mirror then radial (third, fifth and seventh order). For each of the target-present (blue data points) and target-absent (magenta data points) conditions for every symmetry type, RTs and set sizes are significantly linearly correlated (Pearson’s coefficient was in the range: .84 ≤ *r* ≤ .99, with two-tailed *p*-values in the range: .005 ≤ *p* ≤ .016); the data are hence presented with fitted straight lines.

Multiple (Bonferroni corrected) two-tailed *t*-tests were performed on the observers’ search slopes, that is, the gradients of the RT versus set size plots. Considering the target present conditions (correct responses only) the analysis revealed searches for third-order radial symmetric targets to be less efficient that mirror-symmetric targets, *t*(9) = −2.96, *p* < .05. However, an increase in efficiency, that is, the gradient of the RT versus set size plot decreased, was found for fifth-order radial; this increase resulted in equal efficiency to that of mirror symmetry, *t*(9) = .46, *p* > .6. Finally a further increase in efficiency was revealed for seventh-order radial- compared to mirror-symmetric targets, *t*(9) = 4.65, *p* < .001), that is, seventh order radially symmetric patterns were detected more easily than mirror symmetry patterns.

Considering the proportion correct data (Figure 3, bottom row) as the set size increased more targets were missed, as indicated by the blue curves with negative gradients. Additionally, apart from a reduction in accuracy in the third-order radial condition performance was high (∼0.98) when identifying target absent trials, and independently of set size.

## Discussion

Our data show that (a) search times are dependent on the number of distractors, that is, they are serial, consistent with Olivers and van der Helm’s (1998) finding that symmetry detection requires selective attention; (b) as the order of radial symmetry increases, search times decrease; (c) mirror-symmetric patterns are detected with higher efficiency than third-order radial patterns, with equal efficiency as fifth-order radial patterns, and with lower efficiency than seventh-order radial patterns. Thus providing the order is high enough, human observers are as sensitive, or more sensitive, to radially symmetric compared to mirror-symmetric patterns. This is in keeping with the idea that radial symmetry is a visually salient property of objects, important for detecting certain biological objects such as the ones shown in Figure 1. It is also consistent with van der Helm’s prediction that salience increases with the number of symmetry axes (see Figure 7 in van der Helm, 2011).

The holographic model of perceptual goodness is a generic model which aims to predict the saliency of a stimulus based on any repeating patterns it may contain (van der Helm, 2011; van der Helm and Leeuwenberg, 1996); hence, it is potentially applicable to our radial symmetric stimuli. However, when comparing out data to predictions based on the holographic model, we identify some inconsistencies. The holographic model predicts that our seventh-order radial pattern should be less salient that our mirror-symmetric stimuli, whereas we report the opposite. Our data are however consistent with the holographic model if our seventh-order radial stimulus is interpreted as a sevenfold reflection (with a small amount of spatial jitter), as the model additionally predicts that a sevenfold reflection should be more salient than stimuli containing onefold mirror symmetry.

An interesting challenge will therefore be to determine whether the order and purity of radial symmetry in animals and plants is correlated with their need to attract interest, as might be suggested by our results.

Why do search times decrease as the order of radial symmetry increases? As the total number of sectors increases, the number of elements per sector decreases (whist the total number remains constant); hence, as the number of sectors increases, a more pronounced circle is ‘swept’ out. In the limit, the total number of elements equals the number of sectors, that is when there is one element per sector, in which case the elements fall on the circumference of a circle. Alternatively, search times could decrease with the number of sectors because the number of potential false matches between sectors decreases.
